# Richard Siddoway Bagnall (1884-1962), Entomologist

**DOI:** 10.3897/BDJ.11.e106860

**Published:** 2023-07-12

**Authors:** Louise Berridge

**Affiliations:** 1 The Natural History Museum, London, United Kingdom The Natural History Museum London United Kingdom

**Keywords:** Richard Siddoway Bagnall, British entomologist, British industrialist, historical collection acquisition, biography, Thysanoptera, Collembola

## Abstract

This biography describes the life and professional work of entomologist and industrialist Richard Siddoway Bagnall (1884-1962). This work significantly expands on the biographical notes of Laurence Mound in his paper “A review of R. S. Bagnall’s Thysanoptera Collections”.

Bagnall’s life and entomological career is described in detail, including a clarification of his birth date. This biography was written to complement the recent digitisation of Bagnall’s Thysanoptera slides at The Natural History Museum, London, and it is hoped that this biography will be of benefit to future workers upon his material. In addition to Thysanoptera, Bagnall also worked on Collembola, Coleoptera, Myriapoda and other groups.

## Introduction

Richard Siddoway Bagnall (1884-1962) was born into a prominent industrial family from the northeast of England and, throughout his life and career, tried to balance his commitment to his business with his interest in entomology, which brought him much recreation and enjoyment. As a young man, Bagnall was friendly in Edwardian entomological circles and very active in the founding years of the Vale of Derwent Naturalists' Field Club, a workers' recreational natural history group in County Durham. At age 27, Bagnall had the chance to work as a professional taxonomist with the Museum collections at Oxford University, but the pull of his business and the First World War derailed his plans. Bagnall was party to some career controversies, especially regarding the dispersal of his Thysanoptera (thrips) collection in the 1930s, but his reputation allowed him to continue working with insects to some degree until almost the end of his life.

During his career, Bagnall published 577 species-group names and 100 genus-group names for Thysanoptera (Mound 1968, pg 3), writing in more than 120 separate publications (Mound 1968, pg 4). When Mound reviewed Bagnall’s species in 1968, he concluded that 383 of Bagnall’s species and 66 of his generic names were still valid, although some required new combinations (Mound 1968, pg 4). Peter Shaw’s list of UK Collembola shows that 41 of the UK species Bagnall named between 1921-1949 were still valid as of 2015 (Shaw 2015).

The Digitisation Team at the Natural History Museum, London has recently completed the digitisation of the NHM’s ca. 95,000 Thysanoptera slides, including around 5,000 that originate from Bagnall’s collection - of which approximately 1,200 represent his primary or secondary types. This biography was written as part of research into Bagnall's collection and will be followed by a future data paper on Bagnall's types.

## The Bagnall family in Winlaton

Winlaton in County Durham, England had been known for its ironworks since 1690, when Ambrose Crowley III (1658-1713), a supplier of nails to the Navy, set up business in the town. The Crowley family business flourished for about 150 years with the Admiralty being the main customer, especially during the Napoleonic Wars. At its peak, the Crowley firm employed about 1500 people (Bourn 1896, pg 117).

The Crowleys withdrew their business from Winlaton in the early 19^th^ century, causing great economic distress (Lynn 2014). A part of the old Crowley site was bought by Richard Siddoway Bagnall Snr. (ca. 1796-1873) who established a new iron-forging concern in 1822, naming it “R.S. Bagnall and Sons”. Bagnall’s works were on a smaller scale than Crowley’s - in 1871, Bagnall Snr. is recorded as employing 44 men and ten boys (Census 1871) - but he provided much-needed employment. The Bagnall family later expanded their business to a site at Hylton and owned Axwell Colliery at Swalwell (Anonymous 1875, pg 1, cl 1). From at least the mid-1850s to 1902, the Bagnalls were recorded as operating 'High Forge' at Swalwell, a forge powered by water diverted from the Lady's Steps weir on the Derwent (Anonymous 1855, pg 561, Anonymous 1902, pg 6, cl 3).

The Bagnalls were well-regarded socially with Richard Snr. being appointed a Guardian of the Poor for Winlaton Parish in 1853 (Anonymous 1853, pg 5, cl 5). When a slater named John Baty*^1^ was found murdered on the road between Blaydon and Winlaton in 1860, Richard Snr. and his son Richard S. Bagnall, Jnr. (ca. 1835-1878) served on the initial inquest jury, which convened in Winlaton’s Commercial Hotel (Anonymous 1860, pg 8, cls 3-5).

**Figure 1.**
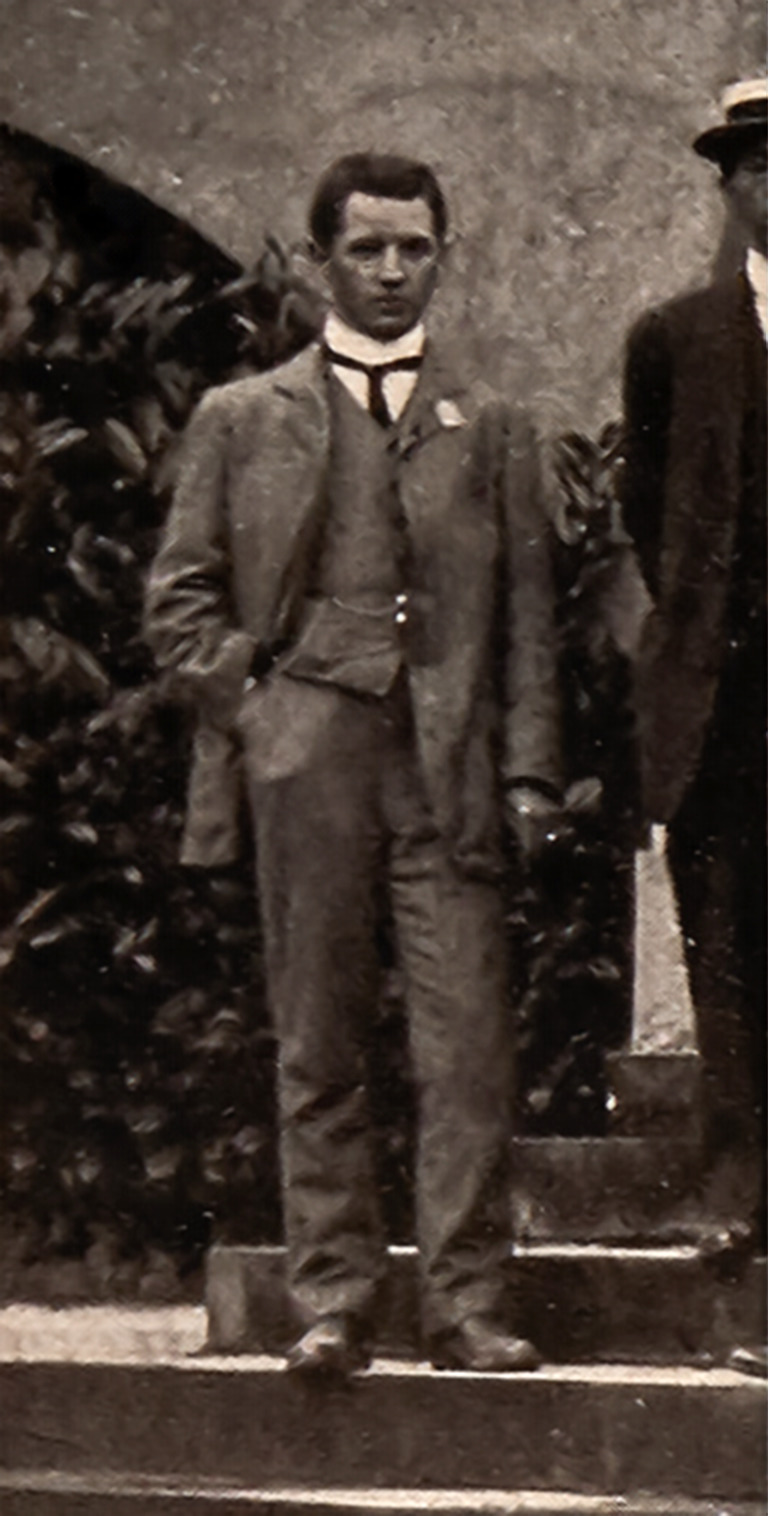
Richard Siddoway Bagnall (aged 26) at the First International Congress of Entomology in Brussels, 1910.

Richard Jnr.’s son, forge master Thomas W. Bagnall (1862-ca.1907), married Emily Florence Lane (ca. 1862-1932) in 1883 and the next year, on 14^th^ July 1884*^2^, their son, the future entomologist Richard Siddoway Bagnall (Fig. 1) was born – along with his twin brother, Charles Lane Bagnall (1884-1974) and followed by a younger brother William Angus Bagnall (1886-1960). The family’s house in Winlaton was named ‘The Groves,’ in North Street in the centre of town (it still stands today, divided into separate properties).

## Bagnall’s early life

Bagnall survived meningitis as a child, but his health did not completely recover and he was described as having a delicate constitution (Anonymous 1962, pg 11) (as an adult, Bagnall makes references to being laid low by rheumatic fever, flu and being "crocked up."*^3^). Sheltered by his family, who educated him privately, young Bagnall’s first zoological interest was in Coleoptera (beetles) and he made the most of his limited mobility by collecting in his local area.

An early paper by Bagnall was on the beetle *Cryptamorphadesjardinsi* [modern combination *Cryptamorphadesjardinsi* (Guérin-Méneville, 1844)], which Bagnall had found in his own house on 18 September 1906: “whilst searching the cellar at home, I found a beetle, easily recognised as something unusual…" (Bagnall 1906a, pg 275). It was indeed a rare occurrence - *C.desjardinsi* had last been recorded in Britain 15 years earlier by Edward A. Waterhouse (1849-1916), who had found an example in London in a bunch of bananas (Bagnall 1906a, p 275).

In their teens, Bagnall’s brothers Charles and William were sent to a boarding school at Cullercoats (Census 1901c), but Bagnall remained at home (Census 1901b). Bagnall would later say in a letter to the zoologist Karl Jordan (1861-1959) that he found his limited education a hindrance as this made it harder for him to formulate Latin names for his described species: - “I find it very difficult to suggest new generic names, having had neither a formal or classical education" (Bagnall 1908b). Bagnall also found it harder to engage with taxonomic literature, for when Jordan sent Bagnall a copy of his book on Physopoda (=Thysanoptera) which was in German, Bagnall thanked him, but mentioned he would need to have it translated (Bagnall 1908c).

## Involvement with The Vale of Derwent Naturalists' Field Club

In 1896, the naturalist Canon Henry Baker Tristram (1822-1906) had helped to set up the Hancock Prize in memory of ornithologist John Hancock (1808-1890), which comprised £5 (equivalent to about £500 in 2023*^4^) to reward essays contributing to observations of natural history in the northeast of England. It was especially intended for people whose opportunity for scientific nature study had been restricted by their circumstances. Bagnall won this prize twice, in 1903 and 1905 (Robson 1915, pg 12). Bagnall shared the 1903 prize with James Caygill, who was a founder member and first President of the Vale of Derwent Naturalists' Field club, where Bagnall is reported as having presented newly-discovered beetles on at least three different occasions in his teens and early twenties (Anonymous 1903, pg 7, cl 3; Anonymous 1904, pg 7, cl 3; Anonymous 1907, pg 4, cl 6).

The Field Club was an egalitarian enterprise, set up so that steelworkers, miners and their families could socialise, study natural history and ramble in the countryside. During the winter months, the Club held indoor meetings at the Cooperative Hall in Rowlands Gill. In the early days, perhaps it was feared that the meetings would be a bit man-heavy as the Club Committee had decided to encourage wider membership by admitting women and boys aged under 16 for free (Henderson 1907). By 1908, the annual subscription was 2/6 for men and 2/- for women (the equivalent in 2023 is about £12.70 and £10 respectively*^4^) (Anonymous 1908, Club Programme).

Bagnall wrote an essay for the Club's Proceedings, "Strangers Zoological", in 1908, which helps to explain why he became an entomologist - it elucidates the shift in perspective that came from studying tiny animals as they made a restricted area seem much larger:

"While rambling in the country I have often thought of the marvellous creatures that are to be found at every step, creatures so small and yet so important and interesting; and I have wondered how many knew them, or, knowing them, gave them a second thought. [...] In simple language I want to show how deeply interesting and engrossing is the study of a life which is but a little obvious to the butterfly-catcher or the birds'-nester; life so small that we shall always require to have our pocket lens; life so little known and yet so full of absorbing interest"(Bagnall 1908a, pg 17).

Bagnall was a regular contributor to meetings and the Club's publications and for 1910-1911, he served as the Club’s President - but he was not the only Bagnall involved: his twin Charles served as the Club's journal editor in 1908 (Anonymous 1908, "Honorary Members") and younger brother William was on the Club Council and Photography Officer (Anonymous 1913b, "Officers"). After an excursion to Whitley Bay and Assembly Rooms on 10 August 1907, the Club members took the time to mourn the passing of Thomas Bagnall, the Bagnalls' father, who had served as Club Vice President and "who always showed a great interest in the welfare of the club" (Adamson 1908, pg 14). Thomas must have been glad of the social opportunities the Club had given his children.

## Entomological contacts and friends

Bagnall was elected a fellow of the Royal Entomological Society of London in 1904 (aged only 19 or 20) and made a fellow of the Linnean Society of London in 1909 (Mound 1968, pg 5). In the first decade of the 1900s, Bagnall carefully made connections with established entomologists. Bagnall wrote to the British Museum’s Entomology Keeper, Charles Owen Waterhouse (1843-1917) in 1905, enclosing a beetle specimen, *Pterostichusparumpunctatus*, he had found at Gibside (Bagnall 1905) [modern combination Pterostichuscristatussubsp.parumpunctatus Germar, 1824, now considered a synonym of *Pterostichus (Pterostichus) cristatus* (Dufour, 1820), this beetle's distribution in the UK is confined to the north of England (UK Beetle Recording 2023)]. This is Bagnall's earliest recorded contact with the Museum and the first of a series of gifts he made of rare, locally-occurring insect species. In 1907, Bagnall wrote asking Waterhouse for permission to examine the British Museum’s specimens of the Thysanoptera suborder Tubulifera - mentioning that, after his father passed away, he had needed to take on more responsibility at the family firm: "I am exceedingly busy at the moment, owing to my father’s recent death & to the Critical state of the Iron Trade – which affects our business (Forgemasters, Chain manufacturers etc.) considerably, but I find a few minutes now and then for Thysanoptera" (Bagnall 1907). Bagnall in 1907 was moving his focus from Coleoptera to Thysanoptera, on which he would first publish in the same year.

**Figure 2.**
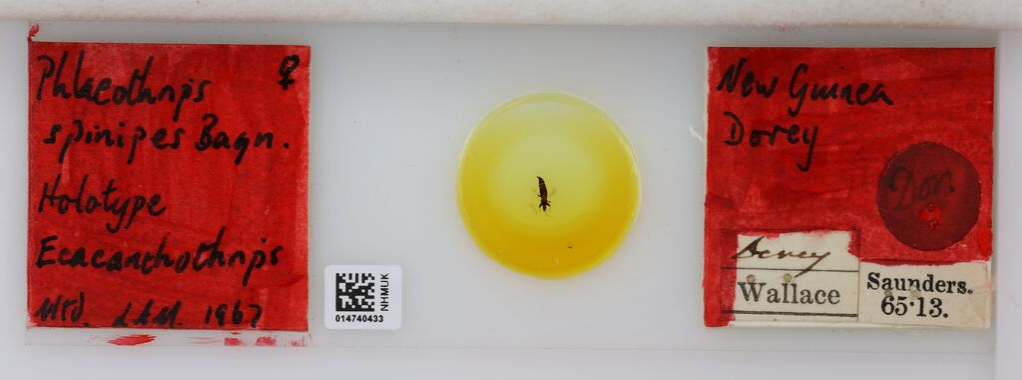
One of Wallace’s specimens from Dorey, examined by Bagnall and described as *Phloeothripsspinipes* [current combination *Ecacanthothripsspinipes* (Bagnall, 1908)]. Formerly glued on to a pinned card, this specimen was remounted for examination by Laurence Mound in 1967 [NHMUK014740433].

While working with the national collection, Bagnall studied specimens collected by Alfred Russel Wallace (1823-1913) that were given to the British Museum by Edward Saunders (1848-1910). Wallace had collected five thrips in Dorey, Papua in 1858. Bagnall seems to have communicated or spoken with Wallace himself about their habitat, as he notes: "Dr Wallace is under the impression that he took these creatures from under bark" (Bagnall 1908d, pg 355) - not bad recollection powers by Wallace of a voyage that had occurred 50 years earlier. Wallace’s own published account of collecting insects in Dorey mentions Elaphomiya (deer-flies) [Saunders' 1861 name *Elaphomiya* is now synonymised with *Phytalmia* Gerstaecker, 1860], beetles and butterflies and he says "insects were tolerably abundant, but were not on the average so fine as those of Amboyna, and I reluctantly came to the conclusion that Dorey was not a good collecting locality" (Wallace 1869, pg 504). Bagnall designated three new thrips species, including one named in Wallace’s honour: *Mecynothripswallacei*, *Macrothripspapuensis* and *Phloeothripsspinipes* [current combination *Ecacanthothripsspinipes* (Bagnall 1908)] (Fig. 2). Bagnall also did not forget to thank Waterhouse - "I would also express my gratitude to Mr C O Waterhouse for [the] kindly help rendered me in examining the collections of Thysanoptera belonging to the British Museum" (Bagnall 1908d, pg 356).

In 1906, Bagnall wrote to the British Museum’s Crustacea Curator William Thomas Calman (1871-1952), enclosing some specimens of the Isopod (woodlouse) species *Trichoniscuspygmaeus*, which Bagnall had found in gardens in Winlaton and in the grounds of the Hancock Museum at Newcastle. Bagnall had been the first to find these woodlice in the UK, after Georg Sars had first described them in 1897 using specimens from Christiania, Norway [Oslo]. Bagnall had asked for help with identifying his own specimens from Alfred Merle Norman (1831-1918) and the Hancock’s Director George Stewardson Brady (1831-1921). Brady knew Sars personally and had been able to ask Sars for Norwegian exemplars of *T.pygmaeus* for Bagnall to examine. (Bagnall 1906b, pg 474).

With a gift for the B.M of his therefore *very* well-provenanced woodlice specimens, Bagnall asked Calman for a small job - "I should be glad if you could let me have a list of the “woodlice” in the British collection, as apart from spp. new to our fauna I may be able to fill in gaps" (Bagnall 1906c). He received a thankful reply: "I noticed your announcement of the discovery of Trichoniscuspygmaeus in the Ann. &. Mag. Nat. Hist. and had intended to write to you to beg the favour of some specimens for the Museum. I was, therefore, particularly glad to get your kind letter and specimens this morning”. Calman went on to say: ‘Our collection of British woodlice is so poor that it is hardly worth while to try to give you a list of them, but as we want to illustrate, as far as possible, the geographical distribution of the species, you cannot send us any – unless, perhaps, *Oniscusasellus* and *Porcellioscaber* – that would not be welcome" (Calman 1906) (the species Calman mentions are common in the UK). Bagnall took this seriously, but searching for British woodlice and ‘filling in the gaps’ took him about five years as he was busy with other work and he finally sent Calman a collection of 28 different British species in April 1911. If Calman were surprised by the long delay, he did not say so, but sent Bagnall a thank-you note: "Dear Mr Bagnall, thank you very much for the valuable collection of British Woodlice which you have been good enough to present to the Museum. Four of the species are new to our collection, and, of the others, a good number have hitherto been represented only by continental specimens, so that your donation forms a most acceptable addition to our series" (Calman 1911).

Bagnall seems polite, but self-assured in his early letters- he knew what he was talking about and could converse with professionals on their level. However, he was leading a double life, cramming entomological study into his free time while his day job was in heavy industry. By adulthood, all three Bagnall brothers were working in the forging business: Richard was a Chain Manufacturer (Census 1911b), Charles was a ‘Forgemaster & Engineer’ (Census 1911a) and William was a Chain Works Manager (Census 1911c). It speaks of a huge amount of personal application that, despite his health problems, Bagnall was essentially conducting two careers at once.

**Figure 3.**
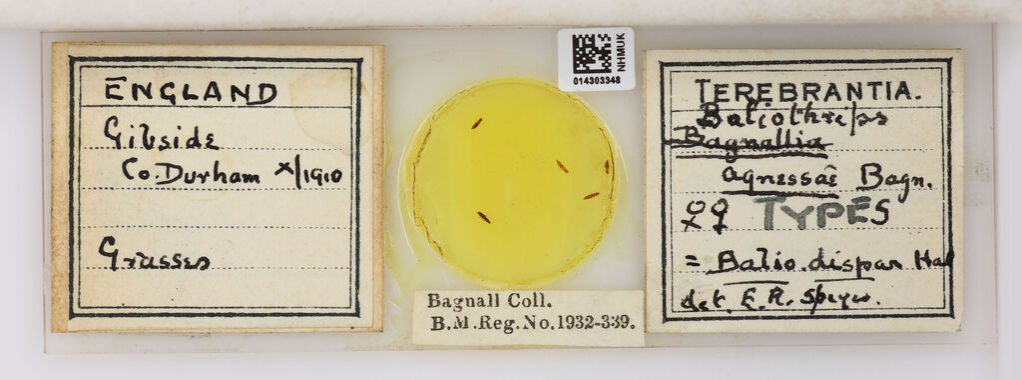
Bagnall's types of his species *Bagnalliaagnessae*, named in 1911, which he afterwards realised was a synonym of *Baliothripsdispar* (Halliday, 1836). The slide label was later amended by Edward R. Speyer [NHMUK014303348].

On 25 August 1909, Bagnall married Agness Allan McIntyre (1886-1974), who was originally from Melrose in Scotland (General Register Office 1909): her family had been living in Winlaton, where Agness’ father worked as an estate manager (Census 1901a) and the couple would later live in Edinburgh. Bagnall is very likely to have named a thrips species he collected from Gibside, *Bagnalliaagnessae* (Bagnall 1911a, pgs 7-8), in honour of Agness in 1911 - although he later realised that it was not a new species and he had accidentally created a synonym for *Baliothripsdispar* (Halliday, 1836) (Bagnall 1914, pg 297) (Fig. 3).

**Figure 4.**
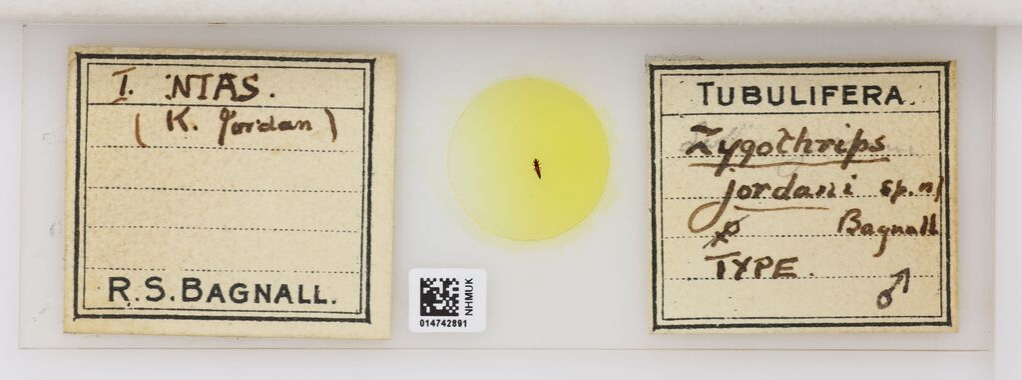
Holotype of *Zygothripsjordani* [modern combination *Haplothrips jordani* (Bagnall, 1909)], described by Bagnall from material sent to him by Karl Jordan and named in Jordan's honour (Bagnall 1909a, pg 530) [NHMUK014742891].

**Figure 5.**
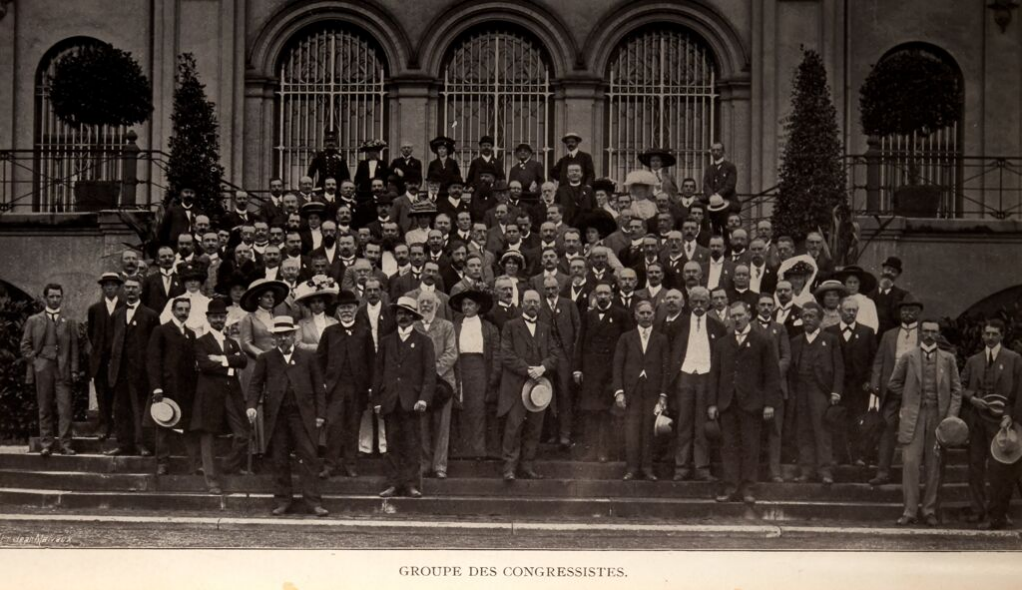
Attendees at the First International Congress of Entomology at the Musee Royal D'Histoire Naturelle, Brussels 1910. Bagnall is standing on the furthest left hand side of the picture. Karl Jordan, Congress organiser, is standing modestly in the middle of the second row from the back. A copy of the Congress publication with a key to identify the participants in the picture can be found at Biodiversity Heritage Library (Severin 1912).

In London sometime around the beginning of 1908, Bagnall had met Karl Jordan, who at that time was working at Lord Rothschild's Museum at Tring (Bagnall 1908b) and they developed a fruitful correspondence. Bagnall expressed his interest in working on exotic Thysanoptera and Jordan had offered him his own collection (Bagnall 1908e), which included material from the Island of Nias in Indonesia (Fig. 4). Around this time, Jordan was working to organise the First International Congress of Entomology (Whitten 2020), which would be at Brussels from 1 - 6 August 1910. Bagnall went to this First Congress, representing the Vale of Derwent Naturalists' Field Club (Bagnall 1913, pg 82) and appears in the group photograph of the attendees (Fig. 5, detail fig. 1).

Some of Bagnall’s other personal relationships can be pieced together from his papers: Bagnall stayed with his friend Horace St John Kelly Donisthorpe (1870-1951) in December 1907 and they collected invertebrates together in the Kew Gardens glasshouses (Bagnall 1908f, pg 428). Bagnall gave millipede specimens (*Brachychaeteumamelanops*) collected from Swanage to the Myriapodologists Stanley (1887-1982) and Hilda (1890-1982) Brade-Birks (Proudlove 2011, pg 8) and they named a millipede variety *Chorduemella scutellare bagnalli* Brade-Birks, 1918 for "our friend and colleague Mr. R. S. Bagnall" (Brade-Birks and Brade-Birks 1918, pg 336) [it is now considered a synonym of *Melogona scutellaris* (Ribaut, 1913)]. In 1915, Bagnall collected Psocids (Booklice or Barkflies) in Whittle Dene with the engineer and entomologist Thomas Hudson Beare (1859-1940) (Bagnall 1915, pg 229) and later would go into an oil venture with him (Bagnall 1919c). Another friend was John William Heslop Harrison (1881-1967), also an iron-worker’s son, who with Bagnall was a co-founder of The Vasculum journal in 1915 which concentrated on the natural history of Northumberland and Durham (Fig. 8). The two men would work together on gall thrips research (Robbins 1993, p 92) collaborating for about a decade (Laing 1937) and Heslop Harrison recommended Bagnall for an honorary doctorate from Durham University in 1929. At the Degree Convocation ceremony, Heslop Harrison presented Bagnall as "a distinguished scientist of Edinburgh" and "the best field worker in the country" (Anonymous 1929a, pg 223). Additionally, in 1929, Bagnall received the honour of being invited to the Entomological Club’s Verrall Supper (Bagnall 1929a).

**Figure 6.**
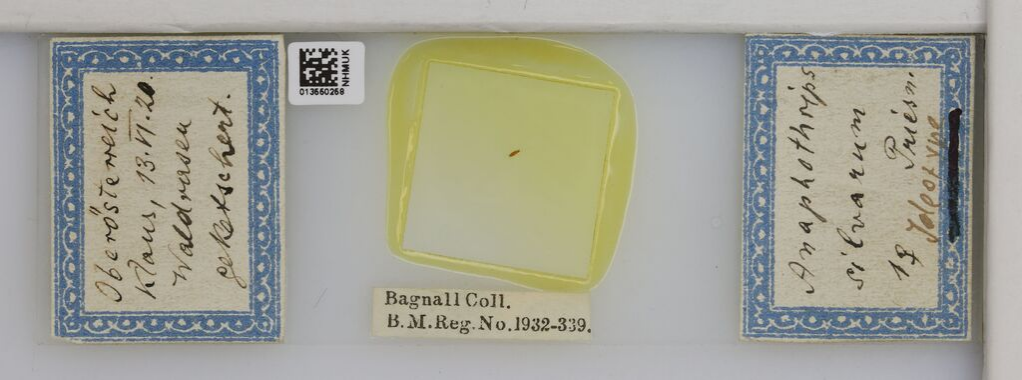
Ideotype of *Rubiothripssilvarum* (Priesner, 1920) from Bagnall's collection [NHMUK013550258].

**Figure 7.**
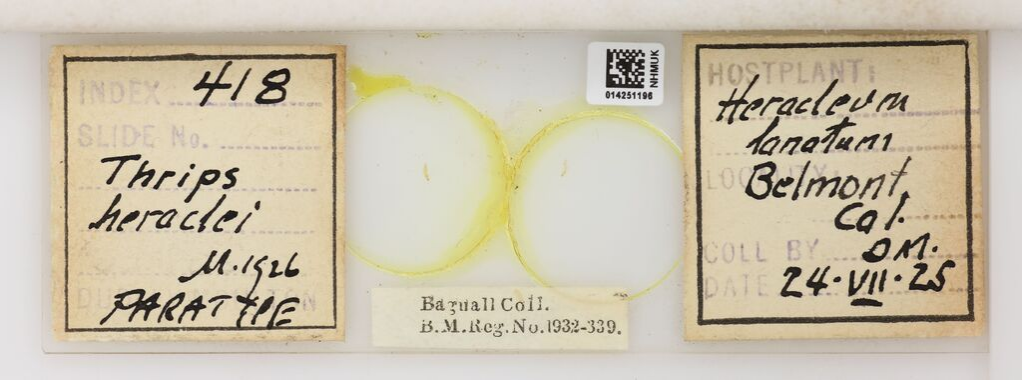
Paratype of *Thripsheraclei* Moulton, 1926 from Bagnall's collection [NHMUK014251196].

**Figure 8.**
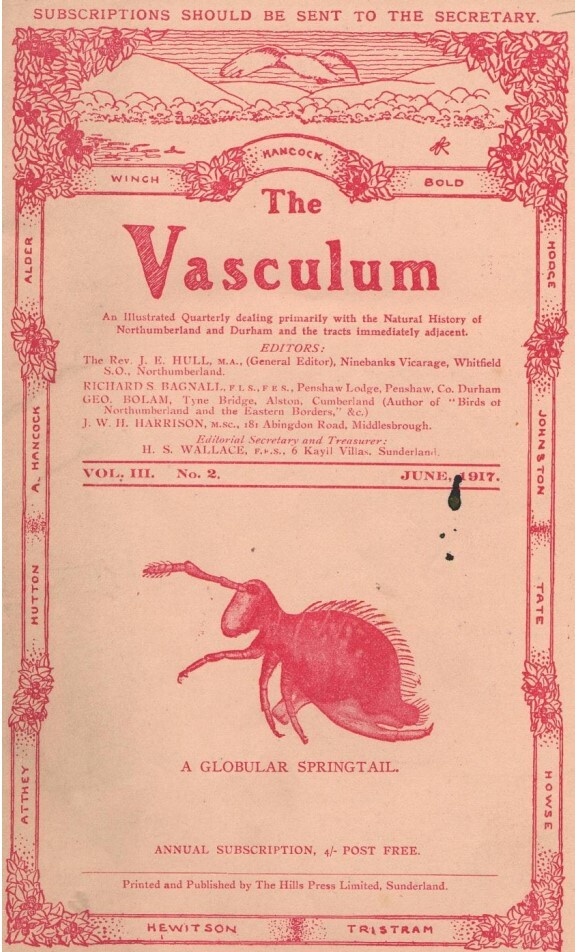
Cover of Volume III, no. 2 of The Vasculum (June 1917) which featured a paper on Collembola by Bagnall (Hull 1917, pgs 52-56).

Of his contemporaries who worked on Thysanoptera, Bagnall favourably regarded Joseph Douglas Hood (USA, 1889-1966), Dudley Moulton (USA, 1878-1951) and Hermann Priesner (Austria, 1891-1974) (Bagnall 1927); Bagnall likely exchanged specimens with Priesner and Moulton, as examples of their material are at the Natural History Museum courtesy of Bagnall’s collection (Fig. 6, Fig. 7).

Perhaps as a reaction to his restricted early years, Bagnall’s adult life was characterised by endless travelling. He went widely around the UK for business and occasionally overseas and would always take the opportunity to look for new insects along the way. In 1908, he visited Belgium and collected thrips in Brussels Botanical Gardens (Bagnall 1909b). He had to go to Norway for business in June 1909 and deliberately came home through Sweden and Denmark to collect and study Thysanoptera and Collembola (springtails) (Bagnall 1911b, pg 60). Bagnall was sufficiently well-off to run a motor car in the early 1920s and went on a motoring weekend to Loch Tay, Scotland in September 1921 (collecting, of course, en route) (Bagnall 1922a, pg 18). Guy D. Morison (1898-1978) noted that Bagnall had at least 17 different addresses between 1904-1949 (Mound 1968, pg 6) – several of Bagnall’s surviving letters are written on hotel stationery. Bagnall leaves the impression of barely sitting still. A negative effect of Bagnall’s itinerance was that he found it hard to access his collection and reference books, sometimes submitting species records with very little comment: "I regret that, living in hotels, away from my literature, specimens, and appliances, it is impossible to make any useful descriptions or remarks" (Bagnall 1922b, pg 176).

Bagnall had begun seriously collecting and researching the Collembola from around 1909, but not having the time to complete his developing paper meant that eventually many of his discoveries had been written up by others - Bagnall wrote in 1921: “Quite a number of my British examples were referable to species not previously known from our country, but not having the opportunity of dealing with these in detail nor of illustrating the various forms, I never published a paper I had in preparation as long ago as 1910-1911. Since then not a few of my novelties have been brought forward by other workers, in most cases, unfortunately, without descriptive or critical notes” (Bagnall 1921, pg 13). Bagnall tried to salvage what he could of this Collembola work by publishing his occurrence data in *The Vasculum*, but more discoveries could have been credited to him if he had only been able to make the time.

## Bagnall at Oxford, 1911-1913

Bagnall's reputation as a brilliant Entomologist was such that, by 1911, he was offered a job at Oxford University, though he entered work in very sombre circumstances.

**Figure 9.**
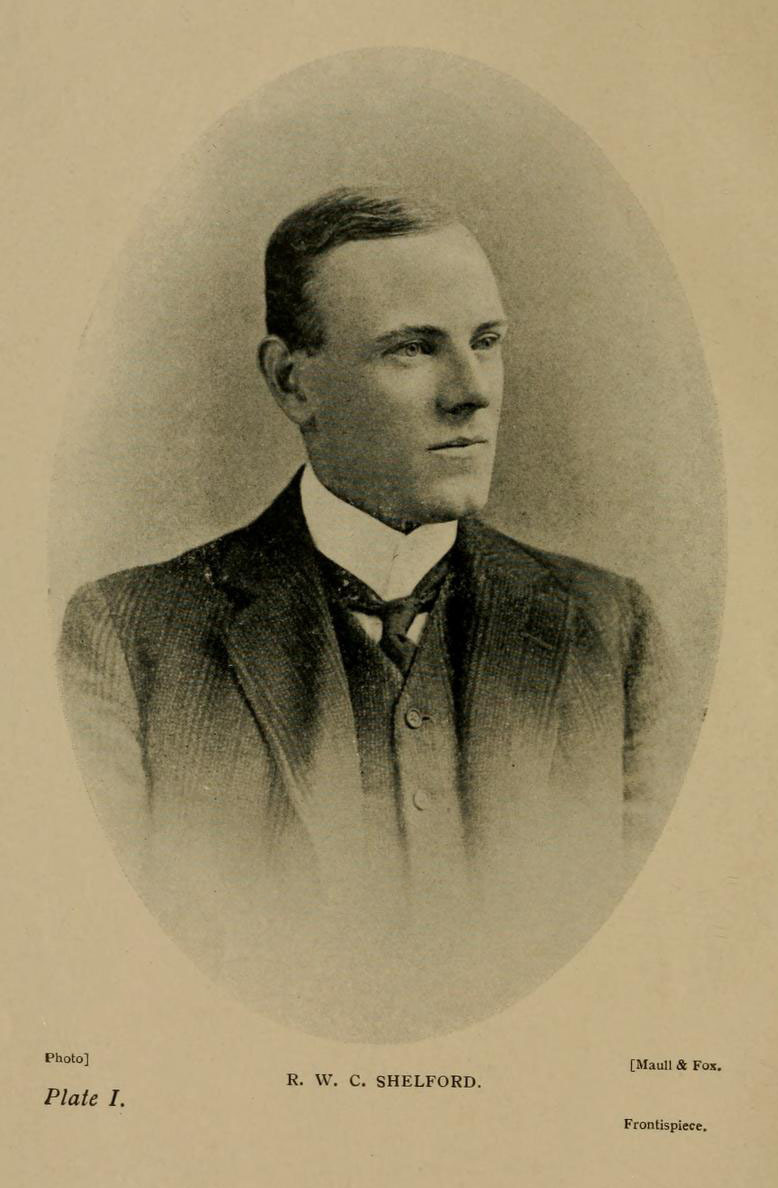
Robert Walker Campbell Shelford (1872-1912), photograph by Maull and Fox (source: Shelford (1916)).

**Figure 10.**
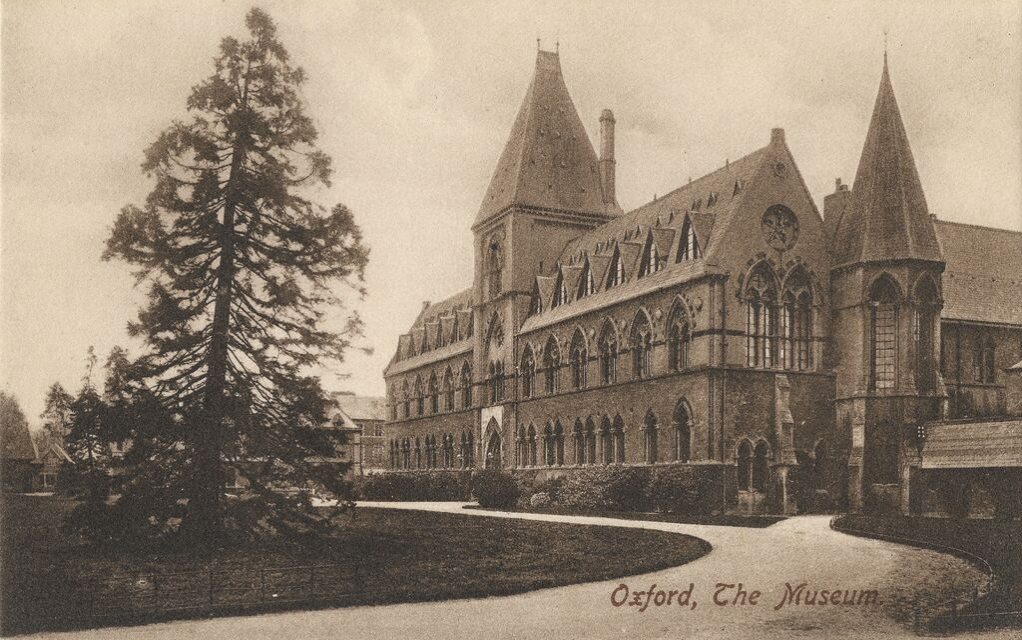
Oxford University Museum of Natural History (ca. 1910), postcard by George Davis of Oxford.

Robert Walter Campbell Shelford (1872-1912) (Fig. 9), assistant keeper at the Hope Department of the University’s Museum of Natural History (Fig. 10), shared an unhappy circumstance with Bagnall: he was another entomologist whose health had been damaged by illness in childhood - for Shelford, tuberculosis affecting his hip after an accident at age three (Poulton 1912a, pg 274). Shelford, like Bagnall, had found solace in natural history after not being able to attend regular school (Poulton 1912a, pg 273). Newly married, Shelford became fatally ill after a fall in April 1909 (Poulton in Shelford 1916, xvii) and although he did his best to continue working, eventually the Hope Department needed to replace him. In 1911, Bagnall was selected as Shelford’s successor (Poulton [undated]) and offered an entomology research position, by the Department head Edward Bagnall Poulton (1856-1943). There is no evidence that Bagnall and Poulton were closely related, despite their sharing a family name (Poulton's mother was named Georgina Sabrina Bagnall (ca. 1819-1894)).

Bagnall did not immediately start work in 1911, having been unable to wind up his involvement with his business. In a letter to Poulton dated 21 March 1912, Bagnall apologises for being unable to register yet: “the work in regard to the Chain Company is taking more time than I thought, partly because of the great care (and in consequence time) we’ve taken in the Constitution of our board & to some extent on account of the lack of faith so many people show & feel in regard to the present Government & on top of that we have this horrible strike” (Bagnall 1912a). This was the National Coal Strike of 1912 in which coal miners were striking for minimum wage, which would have affected the Bagnall family mines. Bagnall wrote to Poulton apologising once again on 28 March – “I am sorry to be forced to humbug you this way, I am however a rapid worker in most things and expect to retrieve lost ground fairly quickly once I am with you" (Bagnall 1912b). Bagnall added that he was hoping to arrive with Agness and set up home in Oxford by the end of April.

Bagnall’s friends at the Vale of Derwent Naturalists' Field Club sent Bagnall warm wishes as he finally left County Durham: ‘Mr R.S. Bagnall, who has been appointed to an important post in the Hope Department of Zoology, University Museum, Oxford, is now no longer in the district, and while his removal is a distinct loss to our Club we tender him our cordial congratulations on his appointment to a post so suited to his great ability. It is with great pride we record the fact that so prominent an entomologist as Mr Bagnall commenced his scientific career with us” (Anonymous 1913a, pg 74). It was later stated by the British Museum's Keeper of Zoology Martin Hinton (1883-1961) that Bagnall was "trained at Oxford, his intention being that he should work for the rest of his life in the entomological department of that University" (Hinton 1942b). Bagnall’s Oxford post seems to have been something that he longed for, which makes events that would occur in the coming years seem particularly sad.

**Figure 11.**
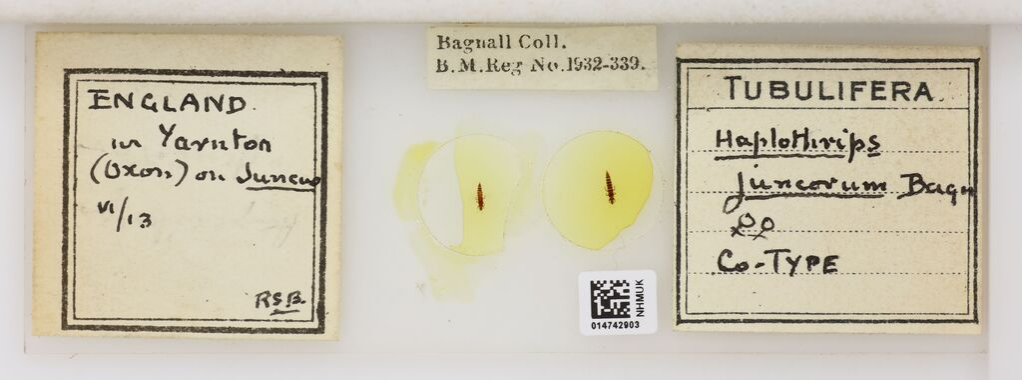
Co-type of *Haplothrips juncorum* Bagnall, 1913, collected by Bagnall in June 1913 at Yarnton, about four miles (ca. 6 km) north of Oxford [NHMUK014742903].

Things began well - Bagnall was able to style himself as of the Hope Department and began writing a paper series "Brief Descriptions of New Thysanoptera" in the Annals and Magazine of Natural History, describing new species using international material that had been submitted to him. Bagnall collected specimens himself in the vicinity of Oxford during this period as his slide labels show (Fig. 11). Bagnall’s new job at the Department sounds like it was fun, with incidents like George Longstaff the Lepidopterist asking the men of the Museum to sniff a mysteriously smelly butterfly *Thaisrumina* [modern combination *Zerynthia rumina* (Linnaeus 1758)] and report their findings back to him - Bagnall reported it was "Musky" (Longstaff 1914, pg 8). Bagnall attended the 1912 International Entomological Congress which was held at the University (Bagnall 1913, pg 82), but did not take part in the impressive group photograph that was taken of the attendees at Wadham College, though the Congress' President Poulton can be seen right at the centre (Jordan and Eltringham 1914).

Bagnall was a notably generous benefactor to the Hope library, contributing copies of his own reprints and papers by others (Poulton 1912b, pg 22; Poulton 1913, pg 54). In 1912, Bagnall made a significant donation of Arthropod specimens with examples of Thysanoptera, Collembola, Chilopoda (centipedes), Symphyla (pseudocentipedes), Pauropoda (tiny arthropods related to millipedes), Diplopoda (millipedes), Isopoda (woodlice), Thysanura (bristle tails), Anoplura (blood-sucking lice) and Mallophaga (biting lice) (Poulton 1913, pg 52). However, Bagnall clearly found it difficult to work at the University for a sustained period of time; in the end, he worked there consistently for about a year between 1912-1913. Poulton summed it up thus: "appointed 1911 but could not begin / also in 1912 he could not begin / at the end of 1913 he had to leave" (Poulton [undated] MS). Bagnall received his last wages from Oxford for the first quarter of 1914 (Mound 1968, pg 6). Poulton did not seek to appoint a successor, due to financial constraints caused by the First World War (Poulton 1923a, pg 2 [68]).

The build-up to the War finally brought Bagnall’s time at Oxford to an end (Poulton 1923a, pg 6). Industrial innovation had become very urgent: in 1913, Bagnall is reported in the Sunderland Daily Echo as forming a syndicate to license the Lelong process, a mechanised method of chain making: "The name of Bagnall has been associated with this ancient industry [chain-making] as far back as the year 1700....Mr R. S. Bagnall, a direct descendant of the founder of the firm, has all along taken a keen interest in the introduction and development of newer processes of manufacture" - the same newspaper page reporting that Zeppelin test flights had been seen over England (Anonymous 1913c, pg 6, cl 5). Bagnall’s brothers were already enrolled in Territorial units (Bagnall 2018; Edwards 2018) and were called to active service when the War began – it is likely that Bagnall’s health meant he could not fight and he ran the family business for the duration of the conflict. As well as ship’s chains, R. S. Bagnall and Sons made castings like engines, stern, rudder and repair frames: in 1918, the firm employed 118 men and 6 women (Anonymous 1918, pg 40). Bagnall was later described as doing "work of national importance at the forges" (Anonymous 1962, pg 11) with Bagnall himself saying in 1917 "I have been engaged upon important Admiralty work since the beginning of the War" (Bagnall 1917); he visited London for appointments with the Admiralty in April 1918 (Bagnall 1919a, pg 81). Bagnall wrote to Poulton on 21 February 1919 mentioning that his twin Charles had just been demobilised and was moving back into his old home (Bagnall 1919c), but even after the War ended, there is no record that Bagnall attempted to regain his old employment at Oxford.

## The dispersal of Bagnall's Thysanoptera collection

Bagnall regarded Poulton highly enough to address him in his letters as "my dear Professor" and they remained in contact, with Bagnall thanking Poulton for getting him through a period of despondency - it sounds like the two were getting on very well: "my little trip to Oxford was a very pleasant one, and I am again indebted to you for real encouragement or should I say – a new supply of enthusiasm when I admit it was much needed" (Bagnall 1919c). In the same letter, Bagnall stated his intention to gift his Thysanoptera collections to Oxford University, getting his solicitors to send Poulton an extract from his last will and testament in which he had outlined his potential bequest:

**Extract from Bagnall’s Will, dated 10 March 1919**: "I give to the Hope Department of Zoology, University Museum in the University of Oxford my collection of microscopic slides of Thysanoptera with the fifty or more cases in which they are contained together with all tubes of material loose slides all loose “seperata” and all bound Volumes on Thysanoptera Myriapoda and Aptera but excluding any cabinets in which the above collection is or may be contained" (Bagnall 1919b).

This Will would not have been legally binding if Bagnall dispersed his collection before his death (as turned out to be the case).

By 1923, some sort of falling-out had happened between the two men which caused Poulton to draft this letter to Bagnall: "I desire you to remember that I do not wish to meet you or shake hands with you and was [only] taken by surprise when I encountered you unexpectedly in the press last Friday evening" (Poulton 1923b). There is no record as to whether Poulton sent this message and to what it referred and it is unfortunately a mystery. In 1925, the two men would cross paths at the Third International Congress of Entomology at Zurich, Switzerland (Bagnall 1925).

In 1926, Bagnall asked Poulton for a contribution towards the costs of preparing his collection: "I am ordering 100 special cases for my collection at 5/9 each (= ca. £15 in 2023*^4^), I wonder if the Department could help towards the cost and also if it would be possible to obtain grants of say £60-80 (= ca. £3,130-£4,173 in 2023*^4^) p.a. towards mounting slides and so free me for the technical work of transcription" (Bagnall 1926). If Poulton agreed to this, it is not unreasonable that he believed this outlay would benefit the Hope Department. Poulton told the Natural History Museum's Director Charles Tate Regan that the Hope Department’s staff had, indeed, helped Bagnall after he left the University: "after he left Oxford a good deal of help was given him by mounting material, the Dept. assistant doing this work & sending him the slides" (Poulton 1932) Fig. 12.

As late as January 1929, Bagnall was still saying that Oxford would receive his collections, but he began to introduce doubt. At first, he mentioned temporarily depositing his collection at the British Museum, with it still going to Oxford later:

"It has worried me rather considerably having in my possession an important collection which might at any time succumb to fire for instance, and on account of the impossibility of being in Oxford or visiting Oxford frequently, I think it would be a good arrangement to house my collection in the new cases I have bought in the British Museum, to be there at my entire disposal and to go to the Hope Dept. upon any later arrangements. / You will very readily realise my difficulties and perhaps you will think the matter over with regard to an arrangement such as this" (Bagnall 1929a).

Two months later, Bagnall told Poulton that he had a preparer working on his slides and: "I will then take the opportunity of making a card catalogue in duplicate and forming my collection in such a way that it is definitely noted as belonging to the Hope Department and housed somewhere in London until such time it is convenient for me to send it to Oxford" (Bagnall 1929b).

By 1932, Bagnall had decided his collection was going to the British Museum and, in an awkward letter to Poulton on 29 November, he first apologised for holding on to some Oxford specimens of Coniopterygidae (dustywings) since the War and then said: "As you know I have found it quite unworkable to have my Thrips collection partly in storage, partly in Edinburgh & partly carried round with me and I have therefore made an arrangement with the B.M. to have it, giving me the fullest access and having the work in progress available for me to take out and work at wherever I happen to be at the moment" (Bagnall 1932). Bagnall did not mention that money was changing hands - the Museum had paid him £285 (Austen 1932)- worth roughly £16,577 in 2023*^4^ . Bagnall promised Poulton a ‘very fine set’ of duplicates for reference, which could not be much of a consolation. "As you are leaving so soon I do not feel so much pleasure in the matter – I should like a chat with you about it…." (Bagnall 1932). Poulton was 79 years old and finally about to retire from the University, only waiting for his successor to be appointed. Bagnall then offered to send Poulton some of his new reprints, which might have been an attempt to follow his bad news with something more pleasant. Bagnall’s letter came late, as Poulton already knew what he was doing, having found out from a British Museum report in his copy of *The Times* newspaper nearly a month earlier, as he told Charles Tate Regan: "I saw in the Times last autumn a statement that he had presented his collection to the Brit. Mus." (Poulton 1932):

**The Times, 24 October 1932**: "The Entomological Department has acquired the collection formed by Dr R. S. Bagnall of the thysanoptera or thrips, notable for their destruction of crops. The 17,000 specimens include 430 types and 750 paratypes, with representatives from most countries where this order of small insects has been observed and collected" (Anonymous 1932, pg 11, cl 2).

## Bagnall's Thysanoptera go to the British Museum

On confirmation that Bagnall had dispersed his collection, Poulton contacted the British Museum to lay out Oxford’s claim to the Bagnall material. Poulton felt that Bagnall's collection, at least that assembled while at Oxford, had only ever been on loan to Bagnall after he left the University: "it was clearly understood that they were the property of the Hope Department and would be returned to it" (Poulton 1932). Poulton went through his own correspondence with Bagnall and underlined every instance in which Bagnall had implied or flat-out stated that his collection would go to Oxford, sending these extracts to the British Museum (Poulton's copies of the correspondence are today kept in the archives at Oxford University Museum of Natural History, including a label by Poulton with red ink: "BAGNALL – SALE OF COLL not his own". It should be said that Poulton’s meticulous record-keeping preserves mainly documents that record what Poulton regarded as the unfair disposal of Bagnall’s collection, as he was trying to build a case against him, so this archive may give a particularly negative impression of Bagnall compared to a fuller and more varied correspondence if it had survived).

A response from the British Museum swiftly came from Norman Denbigh Riley (1890-1979), who had been in post as Keeper of Entomology for only a few months. Riley had joined the Museum in 1911 as an Assistant, 2^nd^ class, in the Department of Zoology (Riley 1964, pgs 1-2). He had volunteered for service in the First World War and rose to the rank of Captain, but had lost two Entomological Department colleagues in the conflict (Riley 1964, pg 9). Riley’s predecessor Major Ernest Edward Austen (1867-1938), the outgoing Keeper, had recommended that the Trustees purchase the Bagnall collection and, as it turned out, Austen had dropped Riley into rather a mess:

**Riley to Poulton**: “I am surprised to hear that you claim some of the types he sold us as the property of the Hope Department. I wish you would thrash this matter out with Bagnall and let us know what conclusion you reach. We naturally assumed that everything offered to us was Bagnall’s property. The published statement that many of the types are in the Hope Department of course can be easily corrected in print. It is well known, I think, that types do not always remain where they were originally placed, and in this case it would only be necessary to publish a note stating where the types are now to be found; the question of real ownership is in rather a different category. As we have already purchased the types, and they are now registered and incorporated, the matter, as far as the Trustees are concerned, is really closed, and it would be extremely difficult to re-open it” (Riley 1932).

However it was not so simple as that, as Poulton was not the only complainant. Bagnall had also sold material that had been loaned to him by Carrington Bonsor Williams (1889-1981) (intended for the Oxford Museum) and specimens belonging to Sir Guy Marshall (1871-1959) (an old friend of Poulton) and the Imperial Institute of Entomology. These items do not appear to be in the Bagnall material at the Natural History Museum today, so it is likely they were returned. Riley had to send an embarrassing memorandum to his Museum Director about the situation and tried to get an explanation from Bagnall.

**Riley to Bagnall**: "The gist of all of them is that you have sold to us material which was in fact not yours to sell, but the property either absolute or in trust, or the parties to whose claims I have called your attention. So that I am in a position to put both sides of the argument before the Director, I should be most grateful if you would be so kind as to let me have a statement of the position as you view it as soon as convenient" (Riley 1933a).

Bagnall’s response to this letter does not survive in the archives. What is left of Bagnall’s explanation is some extracts from it, made by Riley in a letter to Poulton:

**Riley to Poulton**: "I wrote as arranged to Bagnall on January 19th about his collection of Thysanoptera. He duly acknowledged my letter by telephone, but it is only this morning that I have received a letter from him in reply. Although this is headed ‘confidential and without prejudice’, I see no reason why I should not communicate at rate some parts of it to you. // He stoutly denies that any part of the collection he sold to us belonged to the Hope Department. He admits that several of his types are stated to be in the Hope Collection, but goes on to say “this does not affect the question of ownership”. Further he says that “it was understood between Professor Poulton and myself when I took up my duties (in the Hope Department) that any work I did on the Thysanoptera was reserved to me, and the collection was at all times my personal property, and at my entire disposal. The Department did not supply me with material, but when Professor Poulton went to Australia, I persuaded him to collect Thrips for me, and upon his return he kindly presented me with a few tubes of material" (Riley 1933b).

So Bagnall tried to say that Poulton had misunderstood the situation, although it is easy to see why Poulton believed the collection would come to Oxford, considering Bagnall’s earlier statements. It seems that Riley tried Bagnall again, but got a terse response: "Further to yours of 18th February, I have read the accompanying abstracts with great care and I fail to see that any claim to the above collection or any part of it has been established" (Bagnall 1933).

To try to negotiate with Bagnall, Poulton and Riley turned to Bagnall’s old friend John William Heslop Harrison: **Riley to Poulton**: "With regard to Bagnall, my own opinion is that we are very unlikely to get anything out of him unless he is threatened with legal proceedings, but the difficulty about that of course is that your only line of action is against us, as the receivers of stolen property! I hope Heslop Harrison may be able to achieve something for you, although I am not too hopeful" (Riley 1933c).

Heslop Harrison wrote considerately to Poulton, although his letter suggests he was familiar with the evasive behaviour from Bagnall:

**Heslop Harrison to Poulton**: "This Bagnall business seems to get worse and worse. Not the least distressing thing about it is the fact that you yourself are involved in it just when you were retiring and have earned – and earned well - the right to be free from such upsets. // I have seen him [Bagnall] once since last Easter because he never comes to this district now [i.e. Durham, where Heslop Harrison worked at the university] / and I have done no work with him for a long time. However, I shall write to him at the only address I know (9 Charles St London) pointing out all I think about the matter. If anything I can do will afford a solution, I shall be very glad indeed to do it" (Heslop Harrison 1933).

The friendship that was solid enough for Heslop Harrison to recommend Bagnall for his honorary doctorate in 1929 seemed to have cooled somewhat based upon this letter. It is not known what Heslop Harrison said to his old friend, but clearly it was not persuasive. Bagnall’s collection stayed at the British Museum. As Bagnall’s own letter of explanation does not survive beyond Riley’s quotes in the Oxford Archives, it is hard to understand why he decided to burn his bridges with Poulton, which did not seem sensible given that word of Poulton’s distress would quickly spread around the entomological community. Perhaps Bagnall genuinely felt he had made no promises to Poulton and had, therefore, no obligation.

Financial difficulty may have left Bagnall with little choice, but to try to realise money from his collection. The Bagnall ironworks had been propped up by the efforts of WW1, but by the 1920s, R. S. Bagnall and Sons was foundering. Durham Record Office hold the records of the Bagnall firm through several restructurings and consolidations in the 1920s, to the firm’s final liquidation*^5^. In 1925, the Bagnalls sold off their Hylton site to George Slater of Sheffield who shut the works and sold off the equipment for scrap (Anonymous 1925a, pg 4, cl 5). Winlaton would be visited by Edward, Prince of Wales in 1929 as one of the locations in the northeast suffering particular deprivation (Anonymous 1929b, pg 9, cl 4; Anonymous 1929c, pg 1). By 1931, R. S. Bagnall and Sons had ceased to function as a company.

There are indicators that things got bad for the Bagnall brothers as their business failed. Bagnall’s twin Charles sold off his stamp collection in 1923 to try to realise some funds (he was a notable philatelist) (Hanby Holmes (Solicitor) Archives 1923). In 1925, Charles was declared a bankrupt (Anonymous 1925b, pg 7, cl 7) and later prosecuted for continuing to run a business (Anonymous 1937, pg 1, cl 1). In 1930, Bagnall himself was sued along with several other men for taking a disputed loan of £4,500 (= approximately £261,743 in 2023*^4^) from a Mr Cecil Foster while trying to form a company to use the Kalograph Process for printing photographs on metal (Anonymous 1930a, pg 13, cl 4). Bagnall was found not personably liable although the issuing house his company used was found to have acted with ’gross irregularity’ (Anonymous 1930b, pg 11, cl 3). In April 1932, Bagnall’s mother Emily, who had remarried and moved to Italy, died (Alassio Comune Regster Office 1932). It was these circumstances which might have tipped the decision to sell his collection. One can believe that young Bagnall genuinely did intend to gift his specimens to Oxford, but that older harder-headed Bagnall had really needed the money. Whatever the explanation, Bagnall had soured relations with Oxford University for the rest of his life.

Bagnall wrote to Poulton’s successor, Hope Professor of Entomology, George Copley Varley (1910-1983), in June 1955 - more than a decade after Poulton had died - in search of a publication that Bagnall could not track down, knowing that the Hope collection had a duplicate (Bagnall 1955). Varley drafted this response: "Whilst I feel some sympathy for you in your difficulties in getting Stach’s volume, the departmental memory is a long one. Should you be able to make amends for the past, the situation might be different" (Varley 1955).

## Bagnall in later life

Bagnall published his last Thysanoptera paper in 1936 and, from the 1940s, he concentrated on other groups, including going back to the Collembola. Perhaps the controversy from 1932 had dissuaded him from working on thrips. After 1935, Bagnall contributed no more papers to *The Vasculum*, though he did continue to publish in other entomological journals.

In 1937, Bagnall applied for a Leverhulme fellowship to work at the British Museum on a monograph on British galls, to complete his old work with Heslop Harrison. A letter of recommendation was written for Bagnall by Frederick Laing (1890-1965), Assistant Entomology Keeper at the Museum (Laing 1937). This application was not successful *^6^. In 1942 Bagnall was again looking for a research job as the Second World War meant he could not conduct his business as he had lost access to his coal mining interests in Portugal (Hinton 1942b). Bagnall had contacted the British Museum seeking work, but was told the Treasury would be unlikely to fund him (Hinton 1942a) so he tried once again for Leverhulme support, this time with a recommendation from Martin Hinton. This second attempt was successful, with Hinton’s reference which was absolutely glowing: "In my opinion it is a great pity that Dr. Bagnall was ever deflected from his biological work…. I think it would be a thousand pities for Science to allow this opportunity of using a first class worker, of a very rare kind & at the height of his powers, to escape" (Hinton 1942b). Bagnall was awarded £700 over two years to research Apterygota (= approximately £34,989 in 2023*^4^) (Anonymous c.1961). Bagnall and Agness were at this time resident in Harrogate and, while working in London, Bagnall would live in a hotel on the Cromwell Road, not far from the Museum.

Bagnall had intended to work and write on the Symphyla , Pauropoda, Pselaphognatha (a subclass of millipedes) and Chilopoda, hopefully to write a monograph on the first two. He also planned to "acquire material from Correspondents in various parts of the world" (Bagnall 1942b). Bagnall was given use of a room in the Museum’s old Spirit Building and worked with an assistant to help him label up his Thysanura and Collembola material. There is an anonymous report in the Natural History Museum archives, probably by a curator who was assisting Bagnall, alluding to Bagnall’s absences as he had gone out collecting: "a good headway was made in labelling on odd days in February and March 1942 but it ceased when Dr Bagnall went off collecting, and has not since been resumed, despite Dr Bagnall’s frequent attendance at the Museum from the middle of Oct. 1942 to April 1943 and much less frequent attendance up to March 1944 when he ceased to come at all" (this was when the grant money had ended) (Anonymous c.1944).

Whether his research did not prove as fruitful as he hoped or whether other things had got in the way, Bagnall did not end up writing his Monograph. By 1948, Bagnall was wrangling with the Museum to buy his Geophilomorpha (soil centipedes) and Myriapoda slides, but Hinton’s successor Hampton Parker (1897-1968) was reluctant to proceed without the labelling being finished (Parker 1948). This issue had been noted with Bagnall’s Pauropoda in 1942 too: "so much material is inadequately labelled that a large part would be useless if anything were to happen which would prevent you from labelling the stuff for us" (Hinton 1942a). This might have been a reference to Bagnall’s evasive restlessness as much as his age or health. Parker had also tried to persuade Bagnall to remove his remaining belongings that he had abandoned in the Spirit Building in 1942, as the Fish Department needed to use his old office (Parker 1946).

In 1959, Bagnall felt he could do no further useful work with his collections and offered to sell his Collembola and remaining Thysanoptera to the British Museum: "He wishes to sell them. Preferably to us" (Doncaster 1959a). The Museum dispatched a Mr. Carey 200 miles (ca. 322 km) to Harrogate to assess the collection and Dr Teresa Clay was very firm about the value of the material: ‘It would be most unfortunate if this collection containing the types of so many British species was allowed to leave the country" (Clay 1959). So the material, described by John Priestman Doncaster in an internal memorandum as about two cubic feet's worth of slides (Doncaster 1959b), equivalent to a volume of about 60cm^2^, was duly purchased.

Bagnall’s lifelong health complaints worsened until eventually he suffered a series of thromboses (Mound 1968, pg 5) and he died on 19 January 1962, aged 77, at his home in Harrogate. He was survived by his wife, Agness.

## Subsequent work on Bagnall’s collection

In May 1959, Edward R. Speyer (1888-1974) is mentioned as working with Bagnall's Thysanoptera types (Doncaster 1959b). In January 1964, Laurence Mound was appointed to the British Museum’s Entomology Department. Over the next few years, Mound examined Bagnall’s collection and revised Bagnall’s types, resulting in the publication of a comprehensive review of Bagnall’s Thysanoptera species in 1968. Mound noted that Bagnall had a reputation as a "brilliant field worker" (Mound 1968, pg 3) and his esteem of Bagnall’s skill is not overplayed, although he does note the limitations of Bagnall’s approach at times (Bagnall tended to focus on the differences between individual insects rather than study them by population ecology and because of his itinerant lifestyle was never able to produce a synthesis of his own work) (Mound 1968 pg 4). Mound’s work has proved invaluable to the present author for identifying Bagnall’s types on the Natural History Museum’s data portal and for information about the history of the Bagnall collection.

## Figures and Tables

**Figure 12.**
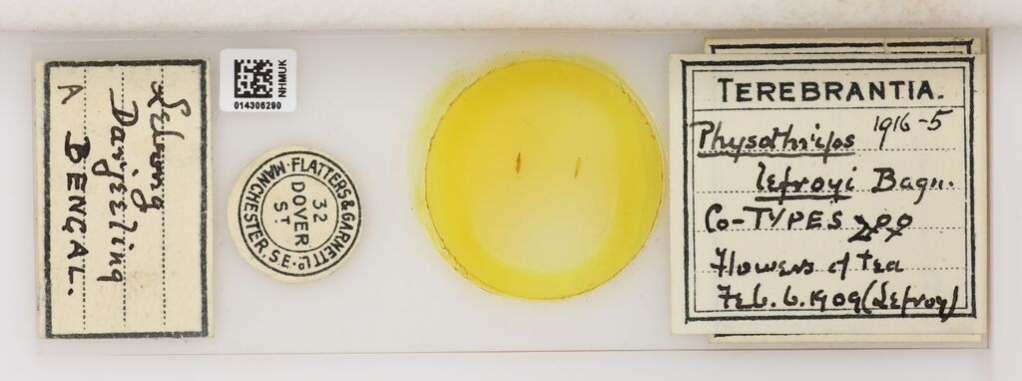
Bagnall sometimes outsourced his slide mounting: the easiest examples to spot in his collection today are slides which were prepared for Bagnall by the firm of Flatters and Garnett of Manchester as these have the firm’s label. Paratypes of *Physothripslefroyi* Bagnall, 1913 modern combination *Lefroyothrips lefroyi* (Bagnall, 1913) [NHMUK014306290].

## Footnotes

*1 In some sources, alternately spelled as John Batey.

*2 Some sources give Bagnall’s birth year as 1889, but this is incorrect. The earlier date of 1884 is confirmed by Bagnall’s birth certificate [General Register Office Ref.: Year 1884: Qtr S: Vol. 10a, page 990] and his given date of birth on the 1939 England and Wales Register [The National Archives; Kew, London, England; *1939 Register*; Reference: *Rg 101/2686b*]. The date of 1889 was supplied to Laurence Mound by Frederick Laing (Mound 1968, pg 5), but it is not clear from where the inaccuracy originated.

*3 References to Bagnall's illness: In a letter to Edward Bagnall Poulton dated 19 May 1926 (Bagnall 1926), Bagnall describes suffering a months-long bout of rheumatic fever, which avoided his heart, but caused arthritis in his wrists and hands with him needing therapy to recover their use. In a letter to Poulton dated 29 November 1932, Bagnall apologies for a delay in communication due to being 'crocked up' (Bagnall 1932). In a letter to Martin Hinton dated 19 March 1942, Bagnall describes having been 'laid up with a 'flu'-cold' (Bagnall 1942a).

*4 For calculating the modern equivalent of historical values, the UK National Archives historical currency calculator (1270-2017) (The National Archives 2017) has been used as it allows historical currencies (e.g. shillings) at intervals of five or ten years to be converted to modern pounds. As the UK National Archives currency converter has not been updated since 2017, the 2017 values obtained from the National Archives historical currency calculator were then applied to the Bank of England's Inflation Calculator (Bank of England 2023) to arrive at an up-to-date value for 2023. The latest Bank of England Inflation figures available were, at the time of writing, dating from May 2023.

*5 The R. S. Bagnall and Co. company records are catalogued at Durham Record Office Archives in the files related to Hanby Holmes Solicitors [ref: Hanby Holmes D/HH 10]. Durham Record Office Archives are currently inaccessible due to relocation, so I have used information, based upon the catalogue listings rather than being able to consult the documents in person.

*6 Leverhulme keep no records for rejected applications from this period so if no record exists, it was not a successful application (A. Grundy pers. Comm).
